# Supplementary material for: Centrioles are frequently amplified in early B cell development but dispensable for humoral immunity
Source: Nat Commun. 2024 Oct 15;15:8890. doi: 10.1038/s41467-024-53222-4 (PMC11480410; doi:10.1038/s41467-024-53222-4)
Supplement: Supplementary file 1 — Supplementary Information [file 41467_2024_53222_MOESM1_ESM.pdf]

Supplementary Information for:

Centrioles are frequently amplified in early B cell development but dispensable for humoral immunity

Marina A. Schapfl[1], Gina M. LoMastro[2], Vincent Z. Braun[1], Maretoshi Hirai[3], Michelle S. Levine[2], Eva Kiermaier[4], Verena Labi[1], Andrew J. Holland[2], Andreas Villunger[1,5,*]

[1]Institute for Developmental Immunology, Biocenter, Medical University of Innsbruck, Innsbruck, Austria

[2]Department of Molecular Biology and Genetics, Johns Hopkins University School of Medicine, Baltimore, Maryland 21205, USA

[3]Kansai Medical University, Department of Pharmacology, Osaka, JPN

[4]Life and Medical Sciences Institute, Immune and Tumor Biology, University of Bonn, Bonn, Germany

[5]The Research Center for Molecular Medicine (CeMM) of the Austrian Academy of Sciences, Lazarettgasse 14, 1090, Vienna, Austria

[*]Address for correspondence:

Andreas Villunger, PhD

Institute for Developmental Immunology

Biocenter, Medical University of Innsbruck

Innrain 80, A-6020, Innsbruck, AT

Ph: +43-512-9003-70380

Fax: +43-512-9003-73960

Email: andreas.villunger@i-med.ac.at

This document includes:
Supplementary Figures 1-8

Supplementary Fig. 1

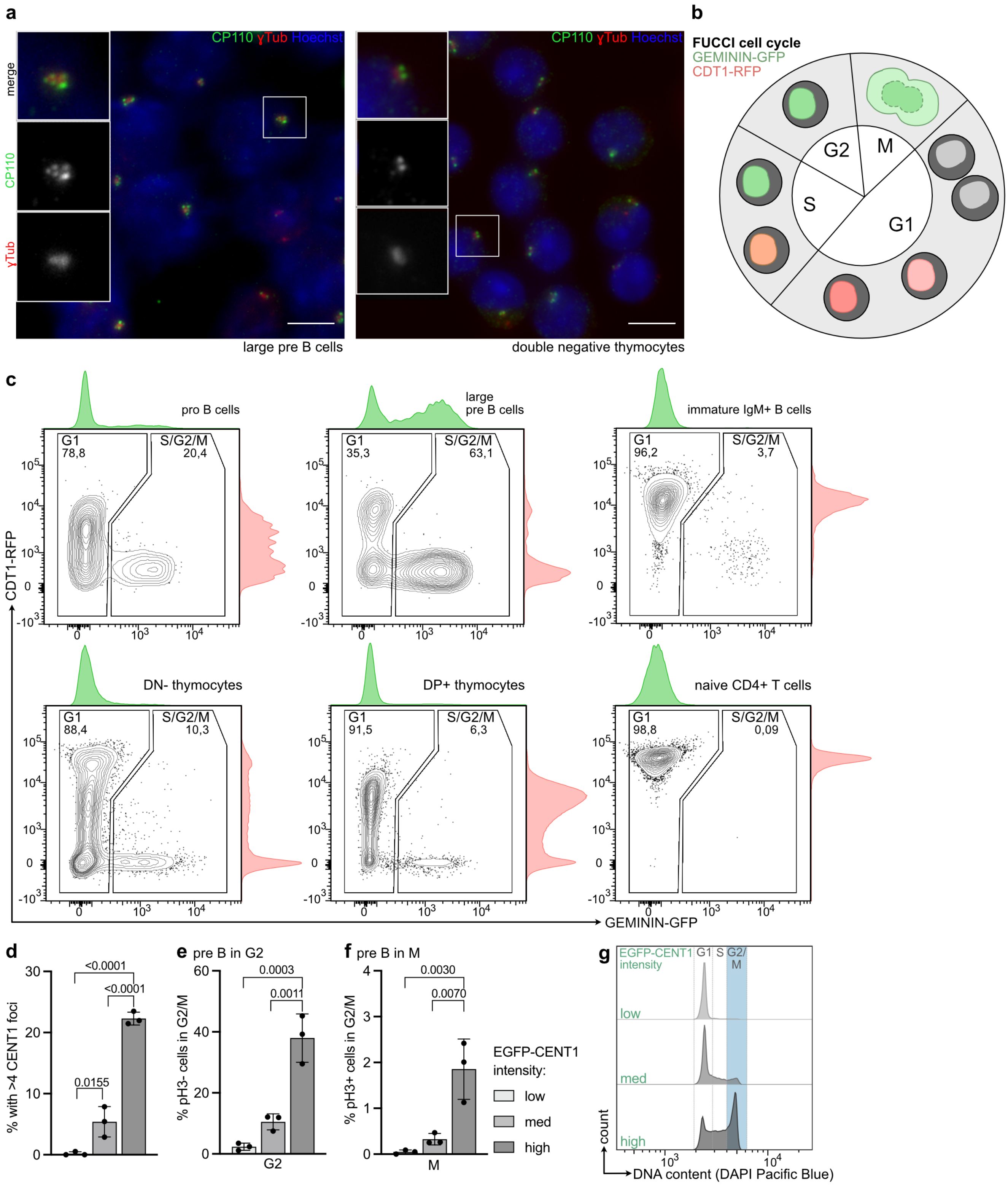

Supplementary Fig 1. Centriole count correlates with proliferative potential in B progenitor cells. a. Immunofluorescence images of large pre B cells (bone marrow) and double negative thymocytes (thymus) of wild-type mice. Cells were stained with CP110, ɣ-Tubulin (ɣ-Tub) and Hoechst. Scale bar, 5µm. **b.** Schematic representation of nuclei labeling in cells isolated from FUCCI mice. **c.** Representative flow cytometry plots (of 3 individual mice, provided with source data) depicting the cell cycle profile of FUCCI mice expressing GEMININ-GFP and CDT1-RFP. Respective cell types are indicated at the top of each plot. Bone Marrow: pro B, large and immature IgM+ B-cells. Thymus: double-negative (DN-) and double-positive (DP+) thymocytes. Spleen: naïve CD4+ T-cells. **d.** Fraction of counted pre B cells with more than 4 EGFP-CENT1 foci was determined by immunofluorescence. Pre B cells from the bone marrow of EGFP-CENT1+ mice were expanded for 7 days on OP9 feeder cells and then FACS-sorted according to EGFP-CENT1 intensity into low, medium (med) and high. n=3; **e.** Fraction of cells in G2 and **f.** M-phase of the cell cycle was determined by flow cytometry. **g.** Representative cell cycle histograms (additional replicates were performed) from sorted pre B cells. Error bars in all panels represent mean ± SD of biological replicates (individual mice). One-way-ANOVA Tukey's multiple comparisons test. Source data and exact p values (<0.0001) are provided as a Source Data file. Graphics in (b) was created using Affinity Designer.

Supplementary Fig. 2

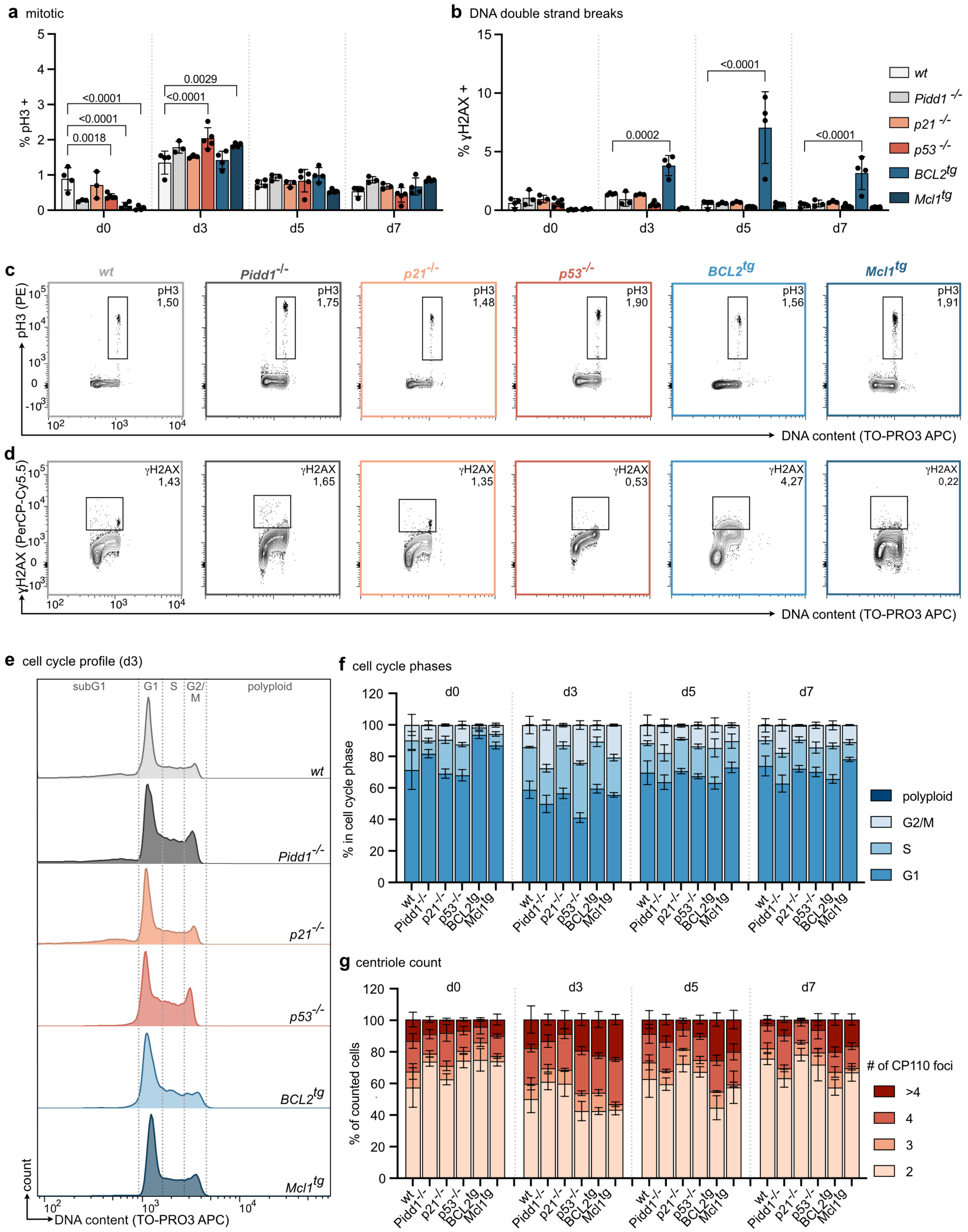

Supplementary Fig 2. Centriole count correlates with proliferative potential in B progenitor cells. a. Pro B cells were FACS-sorted from mice of the indicated genotypes and flow cytometry was used to determine the fraction of mitotic phospho-histone H3 positive (pH3+) cells after sorting (d0) or in culture after 3, 5 and 7 days (d3, d5, d7) with IL-7. wild type *wt* (n=4), *Pidd1*$^{-/-}$ (n=3), *p21*$^{-/-}$ (n=3), *p53*$^{-/-}$ (n=5), *BCL2*^{tg} (n=4) and *Mcl1*^{tg} (n=4); **b.** Bar graph showing the percentage of pro B cells with DNA double-strand breaks (ɣH2AX+). Data are shown as mean ± SD; n-numbers like 2a. **c.** Representative flow cytometry plots (number of replicates is n-number of **a.**) of phospho-

histone H3 (pH3+) positive mitotic pro B cells after 3 days in culture with IL-7 of the indicated genotypes after pregating on viable cells. **d.** Representative flow cytometry plots (number of replicates is n-number of **b.**) of ɣH2AX+ pro B cells, initiating DNA double-strand break repair after pregating on viable cells. **e**. Flow cytometric histograms depicting the cell cycle profiles and gating of FACS-sorted pro B cells after 3 days in culture with IL-7 of indicated genotypes (number of replicates is n-number of **f.**) **f.** Cell cycle profile was determined via flow cytometry directly after sorting (d0) or in culture after 3, 5 and 7 days (d3, d5, d7) with IL-7. Fraction of cells in G1-, S-, G2-/M-phase and fraction of polyploid cells are depicted. *wt* (n=4), *Pidd1*$^{-/-}$ (n=3), *p21*$^{-/-}$ (n=3), *p53*$^{-/-}$ (n=5), *BCL2*^{tg} (n=4), *Mcl1*^{tg} (n=4). **g.** Percentage of cells with 2,3,4 or more than 4 CP110 foci of pro B cells, determined by immunofluorescence with ɣ-Tubulin and CP110 antibody staining and Hoechst nuclear staining. *wt* (n=6), *Pidd1*$^{-/-}$ (n=4), *p21*$^{-/-}$ (n=3), *p53*$^{-/-}$ (n=5), *BCL2*^{tg} (n=4), *Mcl1*^{tg} (n=4). Error bars in all panels represent mean ± SD of biological replicates (individual mice). All genotypes were compared by Two-way-ANOVA Tukey's multiple comparisons test, only comparisons to *wt* are shown (**a-b**). Source data and exact p values (<0.0001) are provided as a Source Data file.

Supplementary Fig. 3

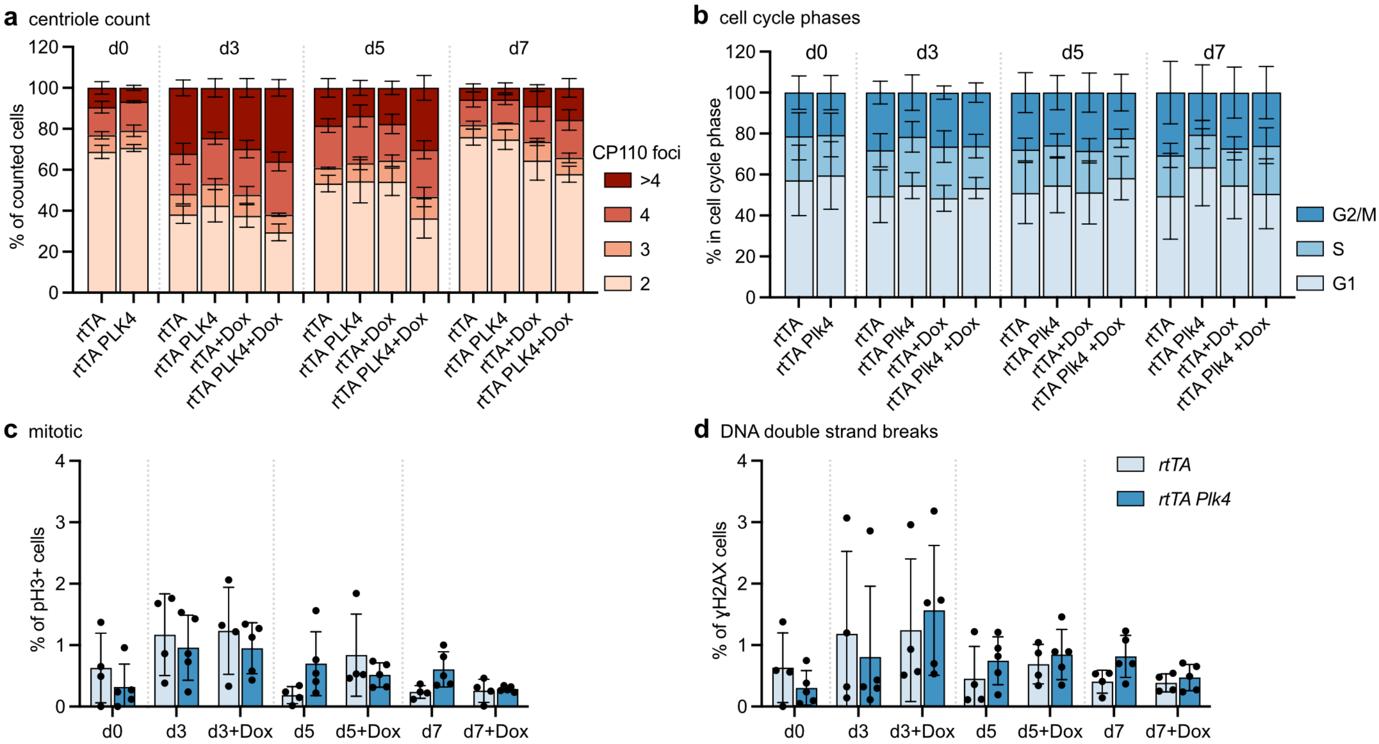

Supplementary Fig 3. Cell cycle profiling and centriole counts in progenitor B cells overexpressing PLK4. a. Pro B cells were FACS-sorted from the indicated genotypes and put in culture with IL-7. Doxycyclin (Dox) was added after 48h in culture. Fraction of counted pro B cells with 2,3,4 or more than 4 CP110 foci were determined by immunofluorescence with ɣ-Tubulin, CP110 antibody staining and Hoechst nuclear staining. n=3 untreated, n=4 *rtTA Plk4* +Dox, n=6 *rtTA* +Dox; **b.** Fraction of cells in G1-, S-, G2-/M-phase of the cell cycle was determined by flow cytometric cell cycle analysis. Data are shown as mean ± SD; n=6; **c.** Bar graphs depicting percentage of cells positive for mitotic marker phospho-histone H3 (pH3+) determined by flow cytometric analysis. n=4 *rtTA*, n=5 *rtTA Plk4*; **d.** Bar graphs depicting percentage of cells positive for DNA damage marker ɣH2AX determined by flow cytometric analysis. n=4 *rtTA*, n=5 *rtTA Plk4*; Error bars in all panels represent mean ± SD of biological replicates (individual mice). Source data are provided as a Source Data file.

Supplementary Fig. 4

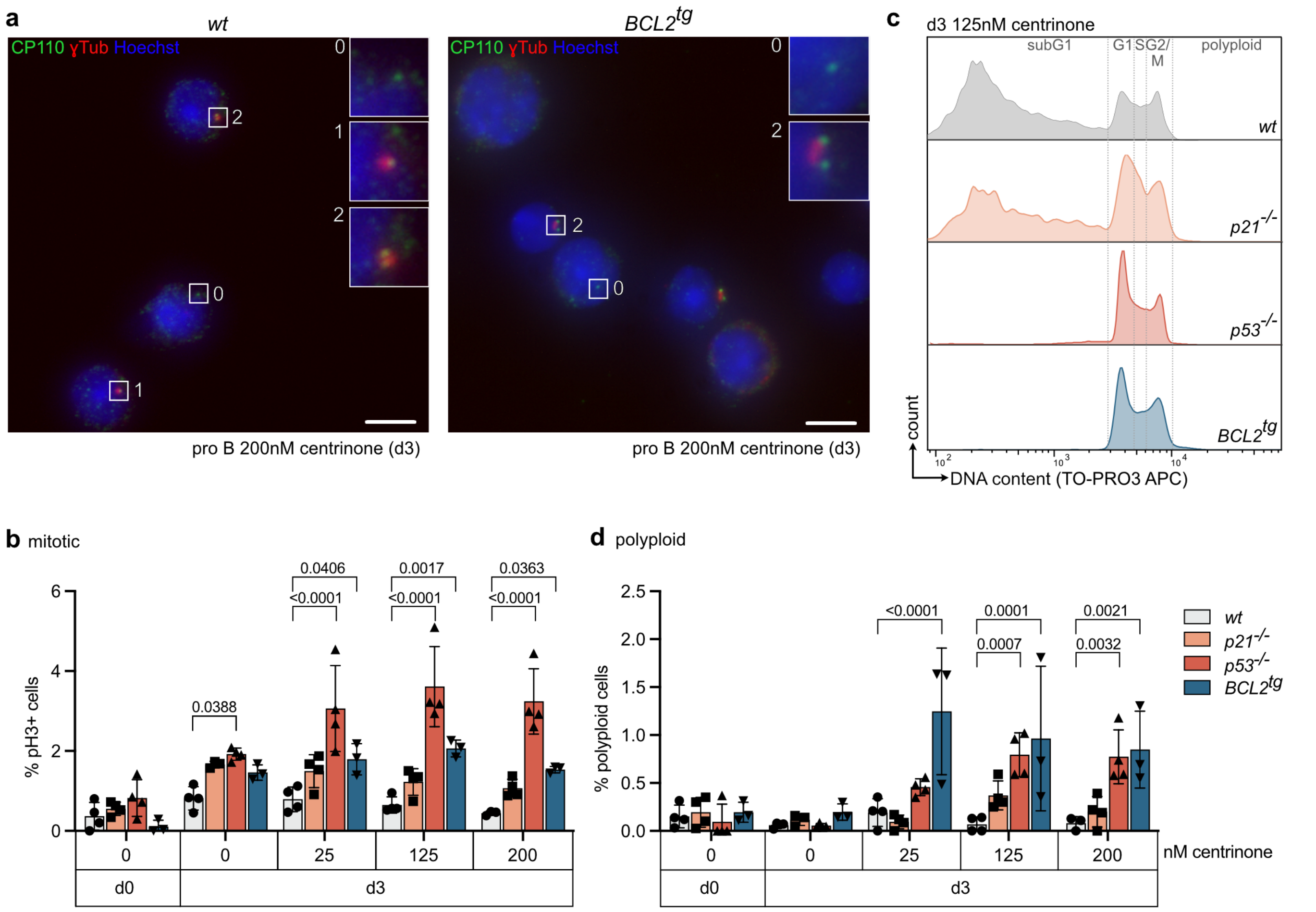

Supplementary Fig 4. PLK4 inhibition depletes centrioles in progenitor B cells. a. Representative immunofluorescence-images of *wt* and *BCL2tg* pro B cells treated for 3 days (d3) with 200nM centrinone stained with γ-Tubulin (γ-Tub), CP110 and Hoechst. Scale bar, 5µM. **b.** Flow cytometric histograms depicting the cell cycle profiles and gating of FACS-sorted pro B cells after 3 days in culture with IL-7 and 125nM centrinone of indicated genotypes. **c.** Fraction of pro B cells positive for mitotic marker phospho-histone H3 (pH3) determined by flow cytometric analysis. n=3-4 for all genotypes; Genotypes were compared to *wt* by Two-way-ANOVA Tukey's multiple comparisons test; **d.** Fraction of polyploid cells was assessed via flow cytometric DNA content analysis. All genotypes were compared by Two-way-ANOVA Tukey's multiple comparisons test, only comparisons to *wt* are shown; Error bars in all panels represent mean ± SD of biological replicates (individual mice). Source data and exact p values (<0.0001) are provided as a Source Data file.

Supplementary Fig. 5

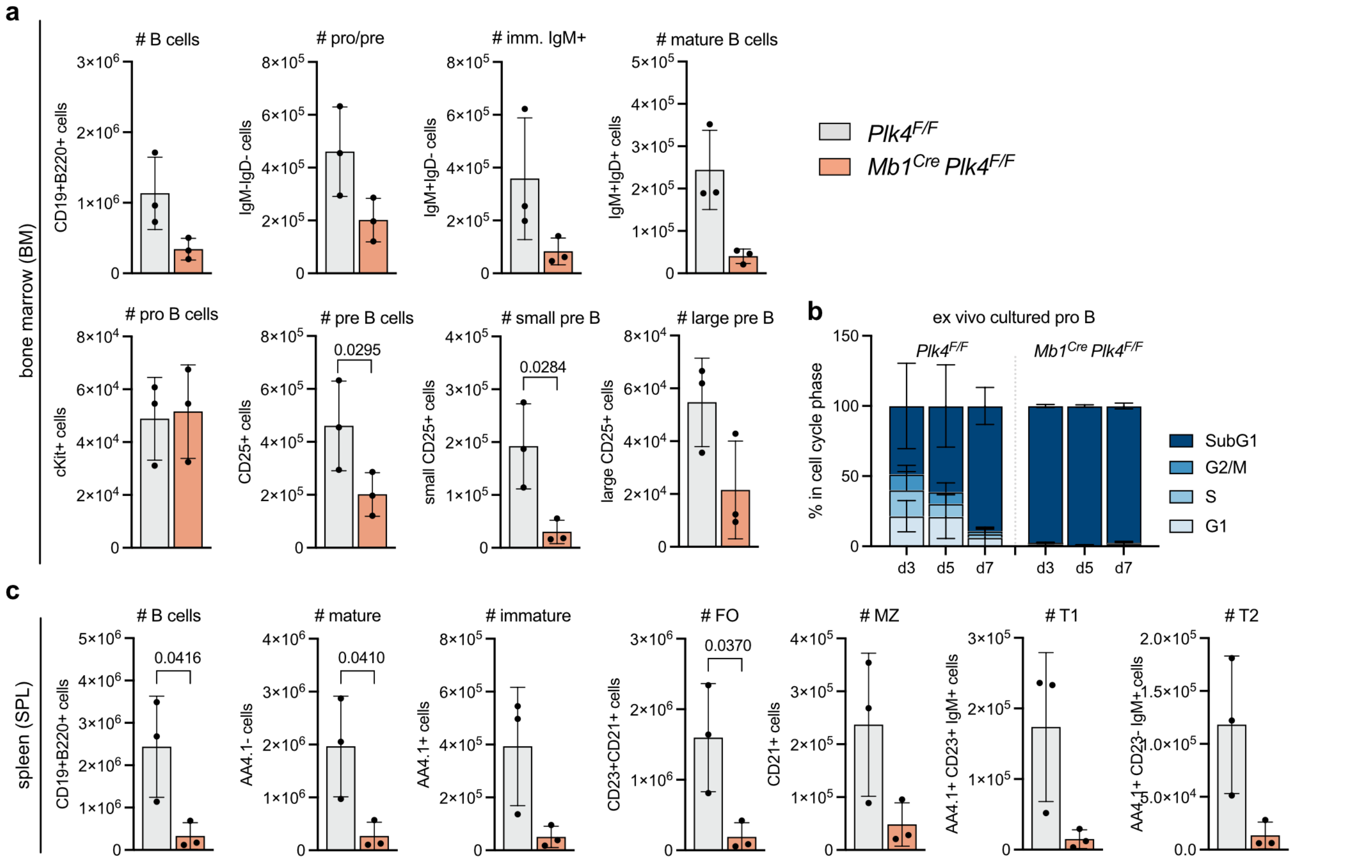

Supplementary Fig 5. Centrosome loss impairs B cell development. a. Total counts of *Plk4*$^{F/F}$ or *Mb1*^{Cre} *Plk4*$^{F/F}$ B cells, pro/pre, immature IgM+ and mature B cells, pro B, pre B, small and large pre B cells in the bone marrow. **b.** Fraction of FACS-sorted pro B cells in G1-, S-, G2-/M-phase of the cell cycle after 3, 5, and 7 days in culture (d3, d5, d7) with IL-7 was determined by flow cytometric cell cycle analysis. **c.** Total counts of *Plk4*$^{F/F}$ or *Mb1*^{Cre} *Plk4*$^{F/F}$ B cells, mature, immature, follicular (FO), marginal zone (MZ), transitional 1 (T1) and 2 (T2) cells in the spleen. Error bars in all panels represent mean ± SD. n=3 biological replicates for both genotypes (individual mice). Compared by two-tailed, unpaired Student's t-test (except mature B cells bone marrow, T1 and T2 were compared by Mann-Whitney test, due to non-normal data distribution); Source data are provided as a Source Data file.

Supplementary Fig. 6

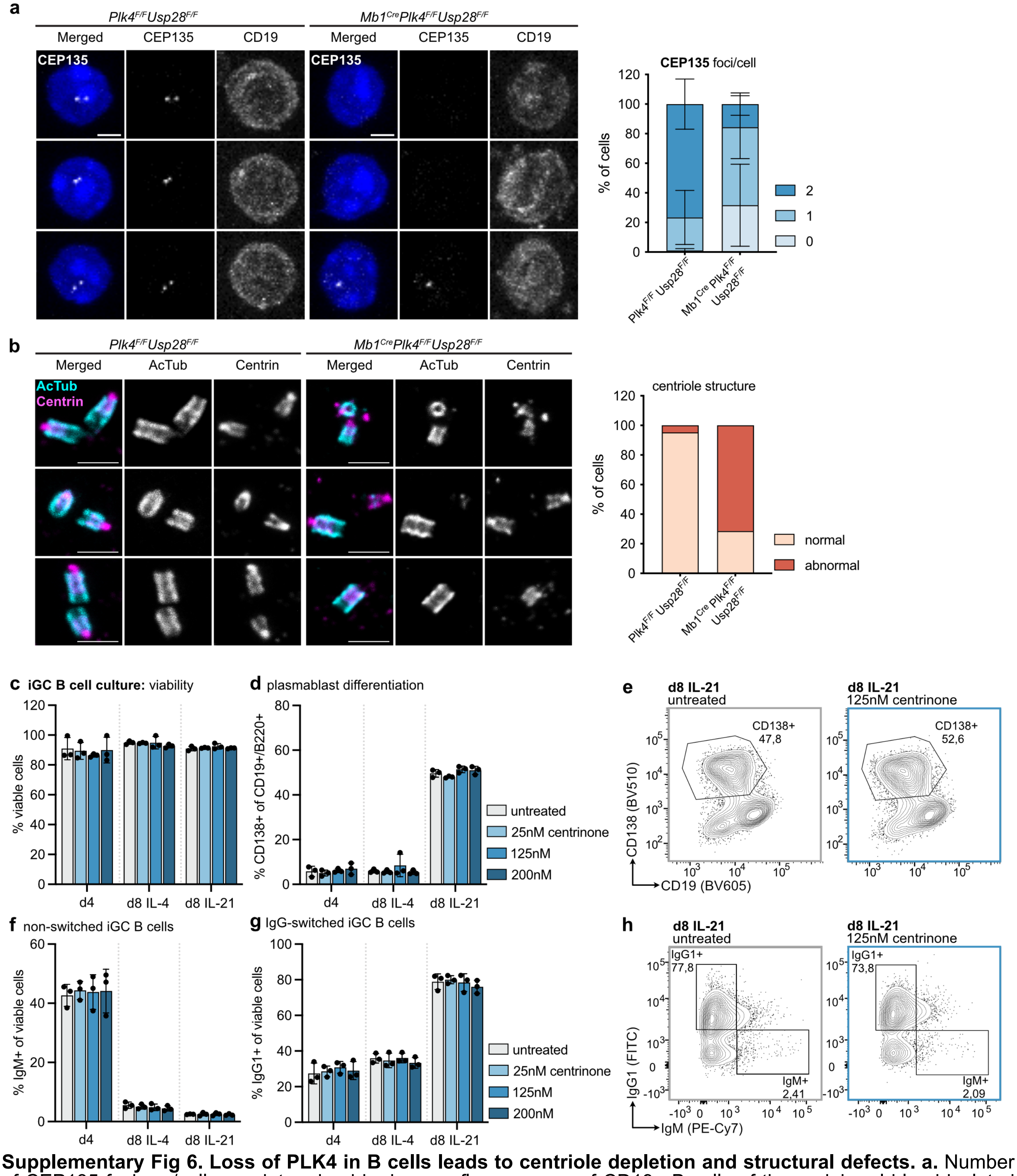

Supplementary Fig 6. Loss of PLK4 in B cells leads to centriole depletion and structural defects. a. Number of CEP135 foci per/cell was determined by immunofluorescence of CD19+ B cells of the peripheral blood isolated from *PLK4$^{F/F}$ Usp28$^{F/F}$* and *Mb1^{Cre} Plk4$^{F/F}$ Usp28$^{F/F}$* mice. Cells were stained with CD19, CEP135 antibodies and DAPI Left, representative images with scale bars representing 5µm. Right, quantification of n=3 individual mice; **b.** Expansion microscopy was used to determine normal and abnormal structure of centrioles in splenic B cells of *PLK4$^{F/F}$ Usp28$^{F/F}$* and *Mb1^{Cre} Plk4$^{F/F}$ Usp28$^{F/F}$* mice. Cells were expanded by a factor of 4 and stained for acetylated Tubulin (AcTub) and centriole marker Centrin. Left, representative images with scale bars representing 500nm. Right, quantification of n=21 cells of 1 mouse; **c.** Flow cytometry analysis of viable (TO-PRO3-negative) B cells cultured on 40LB feeder cells with IL-4 for 4 days, and additional 4 days with either IL-4 or IL-21, treated with graded concentrations of centrinone (25, 125, 200nM). **d.** Fraction of CD138+ plasmablasts within total iGC B cell cultures. **e.** Representative FACS plots (of 3 replicates) showing CD138+ plasmablasts of iGC B after 8 days expansion (4 days IL-4 followed by 4 days IL-21) untreated or with 125nM centrinone. **f.** Fraction of non-switched IgM+ iGC B cells of viable cells. **g.** Fraction of switched IgG1+ iGC B cells of viable cells. **h.** Representative FACS plots (of 3 replicates) illustrating the gating of IgG1+ and IgM+ iGC B after 8 days expansion (4 days IL-4 followed by 4 days IL-21) ± 125nM centrinone. Error bars in all panels represent mean ± SD of n=3 biological replicates (individual mice). Source data are provided as a Source Data file.

Supplementary Fig. 7

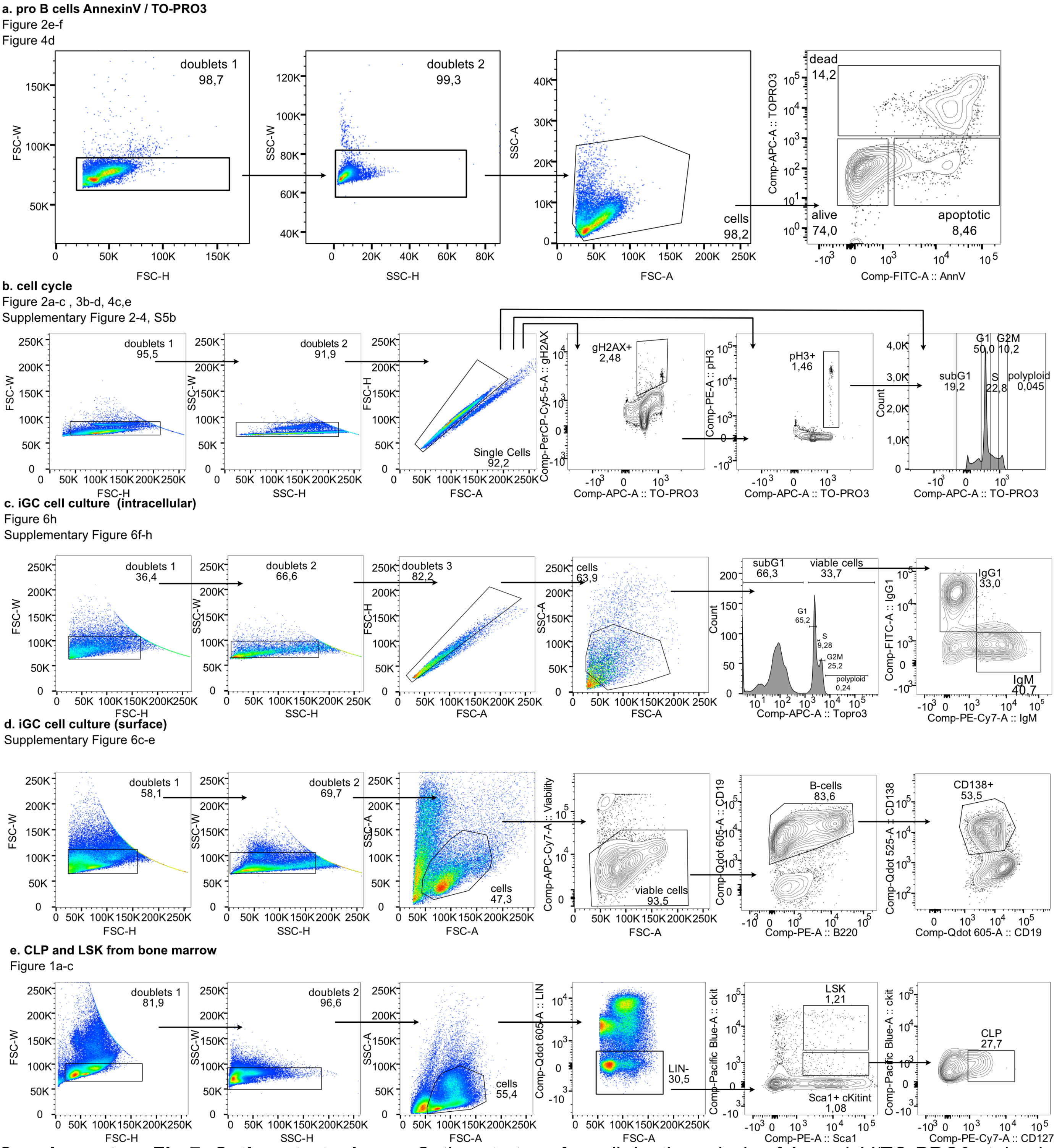

Supplementary Fig 7. Gating strategies. a. Gating strategy for cell death analysis of AnnexinV/TO-PRO3 stained pro B cells. Two times doublet exclusion (FSC-W/FSC-H, SSC-W/SSC-H), gating on all cells (FSC-A/SSC-A) and then gating on viable (AnnV/TO-PRO3-), apoptotic (AnnV+/TO-PRO3-) and dead cells (AnnV+/TO-PRO3+). **b.** Gating strategy for the analysis of cell cycle distrubtion. Three times doublet exclusion (FSC-W/FSC-H, SSC-W/SSC-H, FSC-H/FSC-A) from there gating on mitotic cells (pH3+), cells with DNA damage (ɣH2AX+) and cell cycle (subG1, G1, S, G2/M, polyploid). **c.** Gating strategy of intracellular staining of induced germinal center (iGC) B cells. Three times doublet exclusion (FSC-W/FSC-H, SSC-W/SSC-H, FSC-H/FSC-A), gating on all cells (FSC-A/SSC-A), then gating on cell cycle (subG1, viable with G1, S, G2/M, polyploid), from viable cells forther gating on IgG1/IgM. **d.** Gating strategy of surface staining of iGC B cells. Two times doublet exclusion (FSC-W/FSC-H, SSC-W/SSC-H), gating on all cells (FSC-A/SSC-A) and then gating on viable (fixable Viability dye-), B cells (CD19+B220all). From B cell gate, plasmablast generation was analyzed by expression of CD138+ (CD138/CD19). **e.** Gating strategy for common lymphoid progenitors (CLPs) and LSKs (Lin-Sca1+cKit+). Two times doublet exclusion (FSC-W/FSC-H, SSC-W/SSC-H), gating on all cells (FSC-A/SSC-A) and then gating on Lineage- cells (B220, CD19, CD11b, TCRb, Ter119). From there LSKs were gated by cKit+/Sca1+ and CLPs Sca1+/cKitint and then CD127+.

Supplementary Fig. 8

a. B cell development in the Bone marrow

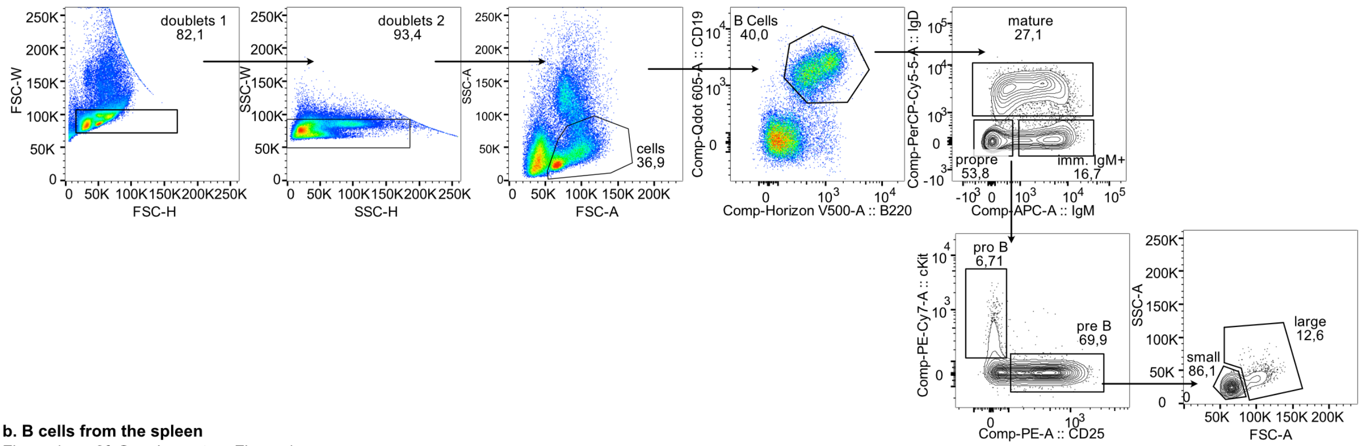

b. B cells from the spleen

Figure 1a-c, 3f, Supplementary Figure 1c

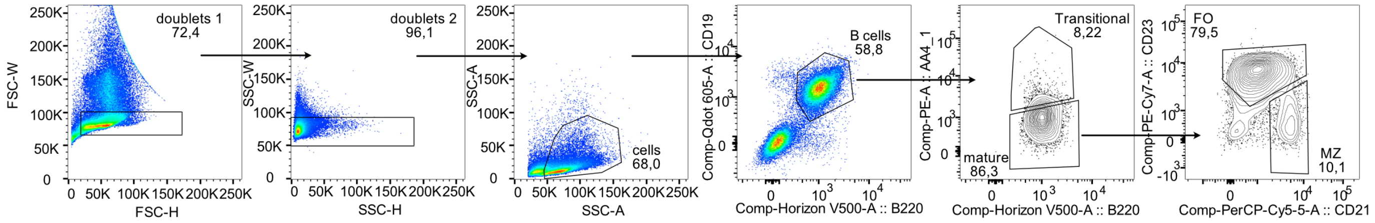

c. B1 B cell from peritoneal lavage

Figure 1a-c, Supplementary Figure 1c

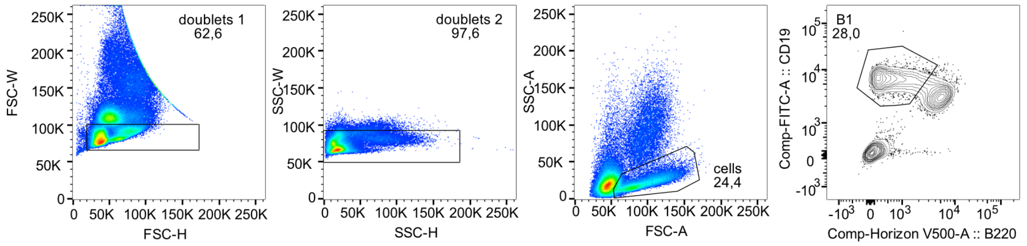

d. T cell development from the thymus

Figure 1a-c, Supplementary Figure 1c

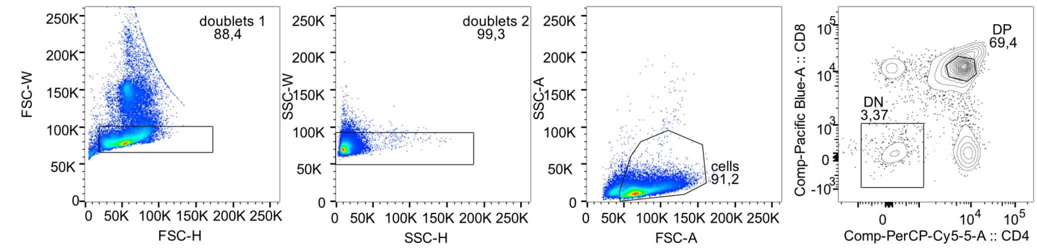

e. T cell from the spleen

Figure 1a-c, Supplementary Figure 1c

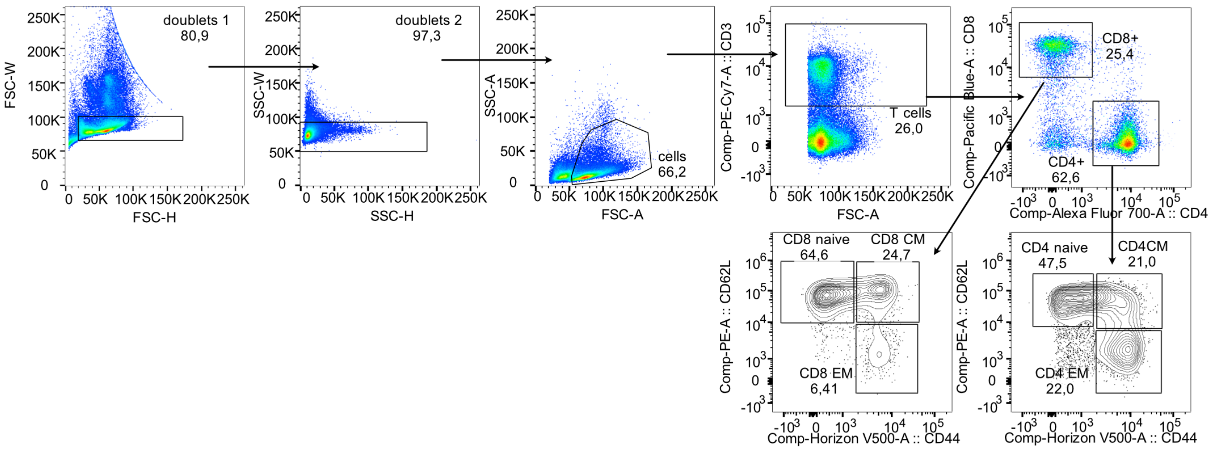

Supplementary Fig 8. Gating strategies. a. Gating strategy for B cell development in the bone marrow, and also sorting of pro B cells. Two times doublet exclusion (FSC-W/FSC-H, SSC-W/SSC-H), gating on all cells (FSC-A/SSC-A) and then gating on B cells (CD19+B220+), from there gating of pro/pre-B (IgD-IgM−), immature (IgD-IgM+) and mature B cells (IgD+IgM+); from pro/pre gate further gating on pro B (cKit+CD25-) and pre B (cKit-CD25+) cells; and from pre B further gating on small and large pre B cells via FSC-A and SSC-A. **b.** Illustrating the gating of splenic B cells. Two times doublet exclusion (FSC-W/FSC-H, SSC-W/SSC-H), gating on all cells (FSC-A/SSC-A), then gating

on B cells (CD19+B220+); transitional (AA4.1+) and mature B cells (AA4.1). From mature B cell gate further gating on follicular (FO, CD23+) and marginal zone (MZ, CD21+CD23-) B cells. **c**. Gating of B1-B cells from peritoneal lavage. Two times doublet exclusion (FSC-W/FSC-H, SSC-W/SSC-H), gating on all cells (FSC-A/SSC-A), then gating on B1-B cells (CD19+B220lo). **d.** Gating strategy of T cell development in the thymus. Two times doublet exclusion (FSC-W/FSC-H, SSC-W/SSC-H), gating on all cells (FSC-A/SSC-A), then gating on double-positive (DP, CD4+CD8+) and double negative thymocytes (DN, CD4-, CD8-). **e.** Gating strategy of T cells in the spleen. Two times doublet exclusion (FSC-W/FSC-H, SSC-W/SSC-H), gating on all cells (FSC-A/SSC-A), then gating on T cells (CD3+); From T cell gate, further gating on CD4+ and CD8+ T cells and from each of them gating on naïve (CD44-CD62L-), central memory (CM, CD44+CD62L+) and effector memory (EM, CD44+CD62L-) T cells.
