# Supplementary material for: Centrioles are frequently amplified in early B cell development but dispensable for humoral immunity
Source: Nat Commun. 2024 Oct 15;15:8890. doi: 10.1038/s41467-024-53222-4 (PMC11480410; doi:10.1038/s41467-024-53222-4)
Supplement: Supplementary file 2 — Reporting Summary [file 41467_2024_53222_MOESM2_ESM.pdf]

nature portfolio

Corresponding author(s): Villunger Andreas

Last updated by author(s): Sep 26, 2024

Reporting Summary

Nature Portfolio wishes to improve the reproducibility of the work that we publish. This form provides structure for consistency and transparency in reporting. For further information on Nature Portfolio policies, see our Editorial Policies and the Editorial Policy Checklist.

Statistics

For all statistical analyses, confirm that the following items are present in the figure legend, table legend, main text, or Methods section.

n/a	Confirmed	
☐	☒	The exact sample size (*n*) for each experimental group/condition, given as a discrete number and unit of measurement
☐	☒	A statement on whether measurements were taken from distinct samples or whether the same sample was measured repeatedly
☐	☒	The statistical test(s) used AND whether they are one- or two-sided<br>*Only common tests should be described solely by name; describe more complex techniques in the Methods section.*
☒	☐	A description of all covariates tested
☐	☒	A description of any assumptions or corrections, such as tests of normality and adjustment for multiple comparisons
☐	☒	A full description of the statistical parameters including central tendency (e.g. means) or other basic estimates (e.g. regression coefficient) AND variation (e.g. standard deviation) or associated estimates of uncertainty (e.g. confidence intervals)
☐	☒	For null hypothesis testing, the test statistic (e.g. *F*, *t*, *r*) with confidence intervals, effect sizes, degrees of freedom and *P* value noted<br>*Give P values as exact values whenever suitable.*
☒	☐	For Bayesian analysis, information on the choice of priors and Markov chain Monte Carlo settings
☒	☐	For hierarchical and complex designs, identification of the appropriate level for tests and full reporting of outcomes
☒	☐	Estimates of effect sizes (e.g. Cohen's *d*, Pearson's *r*), indicating how they were calculated

Our web collection on statistics for biologists contains articles on many of the points above.

Software and code

Policy information about availability of computer code

Data collection	1. FACS data were collected on FACS Aria III or LSR-Fortessa (BD Biosciences) with Software BDFacsDiva Version 9.0.1<br>2. Immunofluorescence images were acquired on Zeiss Axiovert 200M microscope with acquisition software VisiView 4.1.0.3 (maximum stack projections of z-stacks were performed in acquisition software) or<br>3. Expansion microscopy and immunofluorescence images of Fig. 6 and supplementary Fig. 6 were acquired on SP8 (Leica Microsystems) confocal microscope.<br>4. qPCR were performed using StepOnePlus Real-time PCR system (Applied Biosystems)<br>The software used for data collection are also described in “Methods”. No special code were used for data collection.
Data analysis	1. FlowJo version 10.6.2<br>2. Statistical analysis was performed with Graph Pad Prism 10.1.1<br>3. Analysis of immunofluorescence images was performed with ImageJ Version 2.1.0/1.53c by manual counting of foci.

For manuscripts utilizing custom algorithms or software that are central to the research but not yet described in published literature, software must be made available to editors and reviewers. We strongly encourage code deposition in a community repository (e.g. GitHub). See the Nature Portfolio guidelines for submitting code & software for further information.

Data

Policy information about availability of data

All manuscripts must include a data availability statement. This statement should provide the following information, where applicable:

- Accession codes, unique identifiers, or web links for publicly available datasets
- A description of any restrictions on data availability
- For clinical datasets or third party data, please ensure that the statement adheres to our policy

A full data availability statement is included in the manuscript. The information of antibodies, plasmids, qPCR primer sequences and other reagents used in this study were provided in Methods. Source data are provided with this paper. The datasets generated during and/or analysed during the current study are available in the ZENODO repository, under accession code 10.5281/zenodo.10987588, [https://zenodo.org/records/13846975]. Representative FACS plots (fcs files for a minimum of 3 individual replicates) can be found on ZENODO.

Research involving human participants, their data, or biological material

Policy information about studies with human participants or human data. See also policy information about sex, gender (identity/presentation), and sexual orientation and race, ethnicity and racism.

Reporting on sex and gender	N/A
Reporting on race, ethnicity, or other socially relevant groupings	N/A
Population characteristics	N/A
Recruitment	N/A
Ethics oversight	N/A

Note that full information on the approval of the study protocol must also be provided in the manuscript.

Field-specific reporting

Please select the one below that is the best fit for your research. If you are not sure, read the appropriate sections before making your selection.

☒ Life sciences ☐ Behavioural & social sciences ☐ Ecological, evolutionary & environmental sciences

For a reference copy of the document with all sections, see nature.com/documents/nr-reporting-summary-flat.pdf

Life sciences study design

All studies must disclose on these points even when the disclosure is negative.

Sample size	Predetermination of sample sizes with statistical analysis was not performed. Published literature and prior laboratory experience and understanding of expected effect size was utilized to determine the appropriate sample size for each experiment. Samples sizes are reported for each experiment.
Data exclusions	No data were excluded from analysis
Replication	Sample sizes (n numbers) are indicated in the figure legends and correspond to the number of individual mice included in the experimets.
Randomization	The experiments were not randomized. The study does not involve randomized samples.
Blinding	Investigators were not blinded to the group as no human subjects were involved and no subjective measurements were taken.

Reporting for specific materials, systems and methods

We require information from authors about some types of materials, experimental systems and methods used in many studies. Here, indicate whether each material, system or method listed is relevant to your study. If you are not sure if a list item applies to your research, read the appropriate section before selecting a response.

Materials & experimental systems

n/a	Involved in the study
[ ]	[x] Antibodies
[x]	[ ] Eukaryotic cell lines
[x]	[ ] Palaeontology and archaeology
[ ]	[x] Animals and other organisms
[x]	[ ] Clinical data
[x]	[ ] Dual use research of concern
[x]	[ ] Plants

Methods

n/a	Involved in the study
[x]	[ ] ChIP-seq
[ ]	[x] Flow cytometry
[x]	[ ] MRI-based neuroimaging

Antibodies

Antibodies used

Flow Cytometry: Antibody Fluorophore Manufacturer, Catalogue-number, Clone
anti-mouse IgD FITC Biolegend, 405704, 11-26c.2a, 1:100
anti-mouse CD19 FITC Biolegend, 115506, 6D5, 1:100
anti-mouse IgG1 FITC eBioscience, 553443, A85-1, 1:100
anti-mouse CD93 (AA4.1) APC Biolegend, 136510, AA4.1, 1:100
anti-mouse cKit APC Biolegend, 105812, 2B8, 1:100
anti-mouse CD11b APC eBioscience, 17-0112-83, M1/70, 1:200
anti-mouse CD25 APC Bioloegend, 101910, 3C7, 1:300
anti-mouse CD19 APC Biolegend, 115512, 6D5, 1:100
anti-mouse CD62L APC BD, 553152, MEL-14, 1:100
Streptavidin APC Biolegend, 405207, 1:400
anti-mouse IgM (μ-chain) APC Jackson ImmunoResearch, 115-607-020, Daylight/ AF647, 1:1000
anti-mouse CD25 PE Biolegend, 101904, 3C7, 1:200
anti-mouse B220 PE BD, 553090, RA3-6B2, 1:1000
anti-mouse CD93 (AA4.1) PE eBioscience, 12-5892-82, AA4.1, 1:200
anti-mouse IgM PE Biolegend, 406507, RMM-1, 1:100
anti-mouse CD62L PE Miltenyi Biotec, 130-112-836, REA828, 1:200
anti-mouse Sca1 PE Biolegend, 108107, D7, 1:400
anti-mouse phH3 PE Biolegend, 650807, 11D8, 1:200
Strepravidin PE eBioscience, 12-4317-87, 1:400
anti-mouse CD23 PECy7 eBioscience, 25-0232-82, B3B4, 1:100
anti-mouse CD3 PECy7 eBioscience, 25-00331-82, 145-2C11, 1:100
anti-mouse CD127 PECy7 Biolegend, 135013, A7R34, 1:100
anti-mouse B220 PECy7 Biolegend, 103222, RA3-6B2, 1:200
anti-mouse CD117 (cKit) PECy7 Biolegend, 105814, 2B8, 1:200
anti-mouse IgM PECy7 Biolegend, 406514, RMM-1, 1:200
anti-mouse IgD PerCP-Cy5.5 Biolegend, 405710, 11-26C.2A, 1:300
anti-mouse CD4 PerCP-Cy5.5 EBioscience, 45-0042-82, RM4-5, 1:400
anti-mouse CD21/CD25 PerCP-Cy5.5 Biolegend, 123415, 7E9, 1:1000
anti-mouse pH2AX PerCP-Cy5.5 eBioscience, 46-9865-42, CR55T33, 1:400
anti-mouse CD4 APC-Cy7 BD, 552051, GK1.5, 1:100
anti-mouse IgD A700 Biolegend,405730, 11-26c.2a, 1:100
anti-mouse CD21/CD35 A700 Biolegend, 123431, 7E9, 1:100
anti-mouse CD4 A700 Biolegend, 116021, RM4-4, 1:100
anti-mouse B220 A700 Biolegend, 103232, RA3-6B2, 1:400
anti-mouse CD11b A700 Biolegend, 101222, M1/70, 1:200
anti-mouse IgG1 A700 Biolegend, 406632, RMG1-1, 1:100
anti-mouse CD3e A700 BD, 557984, 500A2, 1:100
anti-mouse CD117 (cKit) BV421 Biolegend, 105828, 2B8, 1:200
anti-mouse CD8a BV421 Biolegend, 100738 ,53-6.7, 1:100
anti-mouse CD5 BV421 Biolegend, 100617, 53-7.3, 1:100
anti-mouse IgM eFluor450 eBioscience, 48-5890-82, eB121-15F9, 1:100
anti-mouse B220 BV510 Biolegend, 103247, RA3-6B2, 1:200
anti-mouse CD44 BV510 Biolegend, 103044, IM7, 1:100
anti-mouse CD138 BV510 Biolegend, 142521, 281-2, 1:200
anti-mouse Sca1 BV510 Biozym, 108129, D7, 1:100
anti-mouse CD19 BV605 Biolegend, 115540, 6D5, 1:200
Streptavidin BV605 Biolegend, 405229, 1:400
anti-mouse B220 Biotinylated Biolegend, 103204, RA3-6B2, 1:200
anti-mouse IgE Biotinylated BD, 553419, R35-118, 1:200
anti-mouse CD19 Biotinylated Biolegend, 115504, 6D5, 1:200
anti-mouse CD11b Biotinylated Biolegend, 101204, M1/70, 1:200

anti-mouse TCRb Biotinylated Biolegend, 109204, H57-597, 1:200
anti-mouse Ter119 Biotinylated Biolegend, 116204, Ter119, 1:200
anti-mouse NK1.1 Biotinylated Biolegend, 108704, PK136, 1:200
anti-mouse Gr1 Biotinylated Biolegend, 108404, RB6-8C5, 1:200

Immunofluorescence:
anti-mouse CD19 Alexa Fluor 647 (BioLegend 115522, 1:100)
rabbit polyclonal α-CEP152 (homemade, 1:1000)
goat polyclonal α-ɣ-Tubulin (homemade, 1:1000)
rabbit polyclonal α-CEP135 (homemade, 1:1000)
mouse α-ɣ-Tubulin (Sigma-Aldrich, 1:250)
rabbit α-CP110 (Protein Tech, 12780-1-AP, 1:500)
mouse α-CEP164 (Santa Cruz Biotechnology, sc-515403, 1:500)
mouse monoclonal α-acetylated-α-Tubulin (Cell Signaling Technology 12152, 1:500
rabbit polyclonal α-Centrin (homemade, 1:500)
goat α -mouse IgG AF568 (Thermo Fisher, A11031, 1:1000)
goat α -rabbit IgG AF488 (Thermo Fisher, A-11034, 1:1000)
donkey α -goat IgG AF555 (Thermo Fisher, A-21432, 1:800)
donkey α -goat IgG AF647 (Thermo Fisher, A-21447, 1:800)
goat α -mouse IgG AF647 (Thermo Fisher, A-21235, 1:800)
goat α -rabbit IgG AF555 (Thermo Fisher, A-21428, 1:800)
gaot α -mouse IgG AF555 (Thermo Fisher, A21127, 1:800)

Validation

Validation of flow cytometry antibodies was performed by the manufacturer, and is provided on their websites: https://www.biolegend.com/, https://www.thermofisher.com/at/en/home/life-science/antibodies/ebioscience.html/, https://www.biozym.com/, https://www.jacksonimmuno.com/, https://www.bd.com/en-us/, https://www.miltenyibiotec.com/.

Validation of immunnofluorescence antibodies was performed by the manufacturer, and is provided on their websites: https://www.cellsignal.com/, https://www.ptglab.com/, https://www.sigmaaldrich.com/, https://www.thermofisher.com/, https://www.scbt.com/home/, https://www.biolegend.com/.
Homemade antibodies were previously published and clearly referenced in the text of the materials section.

Animals and other research organisms

Policy information about studies involving animals; ARRIVE guidelines recommended for reporting animal research, and Sex and Gender in Research

Laboratory animals

Laboratory animals included C57BL/6N, C57BL/6J, B6.Pidd1tm1.1, B6.129S7-Trp53tm1Br (p53), B6.Tg(Vav-BCL2)1Jad, B6.Cg-Tg (MCL1) 8Caig, B6;129S2-Cdkn1atm1Tyj/N (p21), B6.Cg-Gt(ROSA)26Sortm1(rtTA*M2)Jae Col1a1tm(tetO-Plk4), B6.TG(CAG-EGFP/CETN2)3-4Jgg,
B6;SJL-Plk4em2Ahol/J Cd79atm1(Cre)Reth and B6;Usp28tm1.1Axbe. Mice were used between 5-8 weeks of age to isolate pro B cells. For other experiments mice were between 10-16 weeks old. Mice were group-housed in a specific pathogen-free facility under standard housing conditions (12-h light/dark cycle, 20-22°C, humidity 40-60%, and free access to water and food).

Wild animals

Wild animals are not used in this study.

Reporting on sex

This study described the findings of both male and female mice (except p53 knock-out animals were only male).

Field-collected samples

This study did not involve samples collected from field.

Ethics oversight

Breeding colonies were approved by the Austrian Federal Ministry of Education, Science and Research (BMWF: 66.011/0008-V/3b/2019), or approved by the Johns Hopkins University Institute Animal Care and Use Committee (MO21M300).

Note that full information on the approval of the study protocol must also be provided in the manuscript.

Plants

Seed stocks | N/A

Novel plant genotypes | N/A

Authentication | N/A

Flow Cytometry

Plots

Confirm that:

- [x] The axis labels state the marker and fluorochrome used (e.g. CD4-FITC).
- [x] The axis scales are clearly visible. Include numbers along axes only for bottom left plot of group (a 'group' is an analysis of identical markers).
- [x] All plots are contour plots with outliers or pseudocolor plots.
- [x] A numerical value for number of cells or percentage (with statistics) is provided.

Methodology

Sample preparation

For FACS-sorting, single cell supensions of spleen and thymus were meshed through 70µm filter, bone marrow was isolated by flushing both femurs and tibiae with a 23g needle and peritoneal lavage was performed to isolate B1 cells. Single cell suspensions were then incubated with 1 µg/mL of aCD16/32 Fc-Block (BioLegend, San Diego, CA, USA, 101310) for 10 min, and then incubated with antibodies of interest.

For AnnexinV/TO-PRO3 analysis of cultured cells, cells were harvested and then stained with Annexin V-FITC (1:1000, Biolegend 640945) and TO-PRO™-3 Iodide 642/661 (1:50.000, Thermo Fisher Scientific, Waltham, MA, USA, T3605) diluted in Annexin V Binding Buffer (1:10 in water, eBioscience, USA 00-0055-56).

For conventional cell cycle analysis cells were fixed in 70% icecold ethanol, permeabilized and then washed with PBS. Cells were then incubated with ɣH2AX (Cell Signaling, Beverley, MA, USA, 2577, 1:400) and phospho-Histone H3 Ser-10 (Cell Signaling 9701, 1:400) antibody in 30 µl permeabilization buffer for 20–30 min. Cells were washed twice with permeabilization buffer, resuspended in 100 µl PBS containing 250 µg/mL RNase A (Sigma R5500), and incubated for 20 min at 37 °C. Finally, 50 µl of 3 µM TO-PRO™-3 Iodide 642/661 (Thermo Fisher Scientific, T3605) or 50 µl 10 µg/mL DAPI (Sigma D9542) in PBS was added.

For the analysis of surface markers of induced germinal center (iGC) B cells, cells were stained with 30ul primary antibody solution. Before acquisition cells were labelled with Fixable Viability Dye eFluor 780 (Thermo Fisher Scientific, 65-0865-14) as per manufacturer's instructions. For intracellular flow cytometric analysis, iGC B cells were washed once with PBS and then treated with trypsin for 10min at 37°C. Subsequently, cells were washed with staining buffer and fixed by the addition of self-made fixation solution (PBS + 4%PFA + 0,1% Saponin) for 20min at 4°C. Cells were washed two times with Perm/Wash (PBS + 1% BSA +0,1% Saponin + 0,025% NatriumAzid) and then incubated with primary antibodies for After washing with perm/wash buffer cells were incubated with second antibody solution (Strep BV605) and after 15min incubation further processed as described in the section intracellular staining and DNA content analysis.

Instrument | LSRII-Fortessa and Aria III (BD Biosciences)

Software | Collection: BDFacsDiva Version 9.0.1; Analysis: FlowJo version 10.6.2

Cell population abundance | The post-sorted population were initially tested by flow cytometry and over 95% purity was achieved.

Gating strategy | The gating strategies are indicated in the supplementary figures 7 and 8.

- [x] Tick this box to confirm that a figure exemplifying the gating strategy is provided in the Supplementary Information.
